# Tau and kappa in interception – how perceptual spatiotemporal interrelations affect movements

**DOI:** 10.3758/s13414-022-02516-0

**Published:** 2022-06-15

**Authors:** Anna Schroeger, Markus Raab, Rouwen Cañal-Bruland

**Affiliations:** 1 Department for the Psychology of Human Movement and Sport, Institute of Sport Science, Friedrich Schiller University Jena, Jena, Germany; 2 Experimental Psychology, Justus Liebig University Giessen, Giessen, Germany; 3 Department of Performance Psychology, Institute of Psychology, German Sport University Cologne, Köln, Germany; 4 School of Applied Sciences, London South Bank University, London, UK

**Keywords:** Spatiotemporal, Kappa, Tau, Interception, Eye-tracking

## Abstract

Batting and catching are real-life examples of interception. Due to latencies between the processing of sensory input and the corresponding motor response, successful interception requires accurate spatiotemporal prediction. However, spatiotemporal predictions can be subject to bias. For instance, the more spatially distant two sequentially presented objects are, the longer the interval between their presentations is perceived (kappa effect) and vice versa (tau effect). In this study, we deployed these phenomena to test in two sensory modalities whether temporal representations depend asymmetrically on spatial representations, or whether both are symmetrically interrelated. We adapted the tau and kappa paradigms to an interception task by presenting four stimuli (visually or auditorily) one after another on four locations, from left to right, with constant spatial and temporal intervals in between. In two experiments, participants were asked to touch the screen where and when they predicted a fifth stimulus to appear. In Exp. 2, additional predictive gaze measures were examined. Across experiments, auditory but not visual stimuli produced a tau effect for interception, supporting the idea that the relationship between space and time is moderated by the sensory modality. Results did not reveal classical auditory or visual kappa effects and no visual tau effects. Gaze data in Exp. 2 showed that the (spatial) gaze orientation depended on temporal intervals while the timing of fixations was modulated by spatial intervals, thereby indicating tau and kappa effects across modalities. Together, the results suggest that sensory modality plays an important role in spatiotemporal predictions in interception.

**Supplementary Information:**

The online version contains supplementary material available at 10.3758/s13414-022-02516-0.

## Introduction

In many daily activities, humans must coordinate their movements both temporally and spatially to intercept a moving object, such as when catching a fly ball. In such situations, temporal and spatial characteristics need to be processed and integrated to act successfully (Fischman & Schneider, 1985; McBeath, 1990; Oudejans et al., 1996; Savelsbergh & Whiting, 1988). In addition, to catch a ball one needs to predict its future location at a concrete point in time. Past research, however, has shown that human perception of space and time is by no means infallible and is sometimes subject to bias. For example, when participants are asked to reproduce the duration of a sound, they show longer reproduction durations when they are holding a long stick between their fingers compared with a shorter stick (Cai & Connell, 2015). A recent review suggests that these interrelations between space and time perception depend on the sensory input, and corresponding differences between visual and auditory information processing in particular (Loeffler et al., 2018). Therefore, the main aim of the current study was to empirically test spatiotemporal interrelations across different modalities in an interception task. To develop and validate a suitable testbed to study spatiotemporal interrelations in interception, in a first experiment, we adapted paradigms of two well-established spatiotemporal illusions, namely tau and kappa effects (e.g., Abe, 1935; Benussi, 1913; Cohen et al., 1953; Gelb, 1914). Thus far, these two phenomena have been mainly investigated in the perceptual domain. In a second experiment, we then further validated and examined differences between the visual and auditory modalities by additionally using measures of predictive gaze behaviors.

### Interception relies on prediction

To start with, actions like catching a ball are typically referred to as interception tasks. They are defined as situations in which one stops the movement of an object by crossing the object’s trajectory at the correct time (e.g., with the hand or a baseball bat). To successfully intercept an object in motion, one needs to accurately plan and execute movements to be in the right place at the right time. Due to sensorimotor delays of 100 ms, this requires predictions of temporal and spatial motion characteristics of the actor, his/her surroundings, or both (Fiehler et al., 2019). Predictions as part of anticipation are based on fundamental perceptual (e.g., visual) and attentional skills (Hodges et al., 2021; Loffing & Cañal-Bruland, 2017). As such, they have been widely studied for visual stimuli, often including eye tracking as a measure of oculomotor processes highly intertwined with motion prediction and interception (Fooken et al., 2021; Fooken & Spering, 2020).

### Spatiotemporal predictions and interrelations

As alluded to above, complex predictions underlying interception are based on perceptual and related processes, including, for example, attention and working memory (Hodges et al., 2021). However, human perception of time and space is far from perfect and can be influenced by other available information. For instance, temporal perception (e.g., the presentation duration of a line) can be affected by spatial information (e.g., the length of the line) and potentially vice versa. It is not surprising that spatial and temporal representations are interrelated when considering that in many situations temporal and spatial features are correlated. Consider the following example: When planning your way to work, two important components to evaluate which route you should take are the distance and the duration. Often, both are associated with each other (the longer the distance, the more time you will need to reach the office), but this association is not necessarily perfect. Some other aspects might play a role as well such as speed or traffic. That means that assuming a strong correlation between time and space may not always be correct and, in fact, may lead to systematic errors—for instance, in anticipating time of arrival based on the distance or vice versa. Assuming strong correlations between time and space might also impact our interception behavior (e.g., when planning where to move on a football pitch; when to grasp for a fly ball; how fast to accelerate one’s own movements). Typically, the higher a juggler throws a ball, the more time she has before catching it. Still, other features can impact the flight duration and might distort her predictions or automatized movements and result in interception errors (e.g., aerodynamic features of different balls).

To conclude, human perception typically relies on the assumption that longer durations come along with longer distance, and consequently it may not be surprising that research has shown that judgements of time can be impacted by spatial information (and potentially vice versa). However, the exact relationship between temporal and spatial representations is not resolved: There is an ongoing debate about whether representations of time and space impact each other reciprocally (symmetrical relationship) or whether spatial representations have a larger influence on how we perceive time than vice versa. The latter notion was proposed in the asymmetry hypothesis (see Casasanto & Boroditsky, 2008; Loeffler et al., 2018; Winter et al., 2015) which is based on the conceptual metaphor theory (CMT; Lakoff & Johnson, 1980). It is assumed that the more abstract representations of time depend asymmetrically on the more concrete spatial representations. This is reflected in language: Spatial metaphors are frequently used to describe temporal aspects, especially in the context of movements (e.g., “The weekend is getting closer”), whereas temporal metaphors are only rarely used to describe spatial concepts (e.g., “I am 5 minutes from the central station”; see Casasanto et al., 2010). Several studies support this theory. For instance, it was shown that the duration of presentation of a line is perceived to be longer with spatially larger lines. On the contrary, when participants were asked to reproduce the length of a line, this was not affected by presentation duration (Casasanto & Boroditsky, 2008; for a preregistered replication, see Whitaker et al., 2022).

On the other hand, another idea about spatiotemporal effects has been put forth, referred to as a theory of magnitude (ATOM; Walsh, 2003), suggesting a symmetrical interrelation. According to ATOM, space, time, and quantities are all processed by a common magnitude system. The core assumption of ATOM is that if all entities share the same neural processing system and consequently attentional and representational resources, there is no reason to expect asymmetrical interrelations between temporal and spatial representations. Instead, it is proposed that both domains impact each other reciprocally. This notion has received empirical support, for instance, by showing that not only judgements of time (duration of a sound) can be influenced by spatial characteristics (e.g., length of a stick), but temporal characteristics can influence spatial percepts as well (Cai & Connell, 2015).

To summarize, both theoretical approaches are supported by empirical studies. While—prima facie—these findings seem to contradict each other, Loeffler et al. (2018) recently suggested that the use of different sensory modalities might explain this discrepancy: Studies supporting an asymmetrical relationship mainly used visual stimuli for both, the spatial and the temporal task, whilst a symmetrical relationship was supported by studies using different modalities (for an overview, see Loeffler et al., 2018).

### Task modality as moderator

Differing sensitivities of modalities explain the discrepancy between ATOM and CMT: The visual system was shown to dominate spatial perception, whereas temporal perception is more dominated by the auditory modality (O’Connor & Hermelin, 1972; Recanzone, 2009). When using mainly visual tasks, as in the studies supporting CMT, representations of temporal aspects of the task might be less precise than spatial aspects. More specifically, introducing the idea of representational noise might shed light on the role of sensory modalities (Cai & Wang, 2021). In several experiments, Cai and Wang (2021) showed that the effect of a context domain on a target domain was modulated by the amount of representational noise (coefficient of variation) within the target domain. If there is more representational noise, the respective dimension is thought to be represented with more uncertainty and might therefore be more prone to influences by the context domain. Applied to the idea of different sensitivities of modalities this means that—because the auditory system is less sensitive toward spatial information—in a mainly auditory task one would expect a spatial representation to be noisier and therefore less stable. Consequently, the spatial representation can be more easily influenced by concurrent temporal information. On the other hand, in a mainly visual setting the temporal representation should be very noisy and therefore prone to be influenced by spatial information. It might therefore be possible to integrate both theories into one model when including task modality in the model’s predictions.

### Tau and kappa effects

Understanding if spatial characteristics affect our perception and prediction of time and potentially vice versa requires disentangling and manipulating time and space independently. A useful testbed for independent manipulations might lie in two perceptual illusion effects, called *tau* and *kappa effects* (Abe, 1935; Benussi, 1913; Cohen et al., 1953; Gelb, 1914; Helson & King, 1931). Previous research has already identified these effects as promising tools to test ATOM against CMT (Alards-Tomalin et al., 2014; Reali et al., 2019).

The *tau effect* is described as the impact of temporal intervals (“context”) on spatial judgements (“primary judgement”). Benussi (1913), for example, asked participants to give a relative judgement about one of two spatial intervals built through the presentation of three successive lights (one interval between stimulus 1 and stimulus 2 and one interval between stimulus 2 and stimulus 3). Results showed that the relative judgements about space (e.g., “the second interval was smaller”) changed with the duration of the two intervals: the interval with the longer duration was judged to be spatially larger. The opposite effect, initially denoted as *S*-effect (Abe, 1935) and later called *kappa effect* (Cohen et al., 1953), illustrates the influence of spatial information (“context”) on temporal judgements (“primary judgement”). In a typical paradigm, participants sit in a dark room and are presented with three successively illuminating lights. They are then asked which temporal interval was longer, the one between the first and second or second and third stimulus. Typically, participants chose the interval with the larger spatial distance between the lights to have the longer duration.

These findings were conceptually replicated and extended by the use of visual, tactile, and auditory stimuli (Helson & King, 1931; Scholz, 1924). In addition, further evidence for *tau* and *kappa* effects was presented for different tasks including, for instance, category judgements instead of relative judgements (Jones & Huang, 1982), reproduction paradigms (Price-Williams, 1954) and memory tasks (Sarrazin et al., 2004; Sarrazin et al., 2007). Together, we deem the *tau* and *kappa* paradigms suitable testbeds to study spatiotemporal interrelations, if appropriately adapted for interception.

### Eye movements

One way to further bridge the gap between mere perceptual processes—as investigated in *tau* and *kappa* paradigms—and interceptive actions may be offered by eye movement research. As mentioned before, eye movements have not only been found to be functionally highly related to motion prediction and perception (e.g., Goettker et al., 2018; Schütz et al., 2011), but hold behaviorally strong associations to interception as well (e.g., Goettker et al., 2019; Mann et al., 2019; Spering et al., 2011). Tracking errors of the gaze are highly related to interception errors (Fooken et al., 2016). Predictive eye movements to future target locations show anticipation of motion trajectories (Mann et al., 2019). It was shown that eye movements (pursuit) are based on perceived rather than actual target motion and consequently biases found for perception are often reported in tracking movements of the eyes, too (cf. Schütz et al., 2011). Perception and pursuit share a common initial motion processing phase and later split in separate pathways (Schütz et al., 2011). As such they are a useful tool to investigate the underlying processes of interception and fill the gap between the two perceptual spatiotemporal interactions in a new action paradigm using an interception task: If effects are absent in the interception data, eye-tracking data might indicate whether this highlights the dissociation between perceptual and action processes or whether the newly developed paradigm is not appropriate to trigger spatiotemporal biases.

### Current study

The aims of the current study with two experiments were twofold: First, we tested whether spatiotemporal (perceptual) illusions, called *tau* and *kappa* effects can impact interception performance. Second, it was analyzed whether there are differences between sensory modalities with auditory tasks strengthening the effect of temporal characteristics on spatial interception (*tau* effect) and visual tasks supporting the effect of spatial characteristics on temporal processing (*kappa* effect). Additionally, in an exploratory manner we tested for contributions of manipulations of the visual and auditory input (blur and volume). To test these hypotheses, in two experiments participants were presented with four successively appearing and disappearing dots or sounds to make them intercept the predicted fifth location at the predicted time of appearance. The first experiment served to test for effects in interception. In Exp. 2, besides replicating the interception results of Exp. 1, gaze data were used to (i) validate the new *tau* and *kappa* paradigm of motion prediction, (ii) address the role of stimulus repetition, and (iii) answer the question whether the dissociation between perception and action might explain absent or unexpected effects.

## EXPERIMENT 1

In Exp. 1, interception data (location and moment of tap) was analyzed to identify *tau* and *kappa* effects in an action task. Based on the low sensitivity of the auditory system to spatial information, it was hypothesized that the interception location would be increasingly overestimated in movement direction with increasing temporal intervals between target presentations, when stimuli were presented auditorily (*tau* effect). In contrast, as the visual system is highly sensitive to spatial and potentially lesser so toward temporal information, this should result in delaying interceptions with increasing spatial intervals (*kappa* effect). The opposite effects for each modality should be smaller or even absent due to the different sensitivities.

### Methods

#### Participants

A total of 43 participants (17 male, *M*_Age_ = 24.2 years, *SD*_Age_ = 3.3 years, sample size similar to previous studies on interception, e.g., Schroeger et al., 2021) took part in the experiment. All provided informed consent prior to participation. Participants had to take part in a vision (Bach, 1996, 2006) and a hearing test (Cotral, Version 1.02B) prior to participation. A minimum visual acuity of 0.00 logMAR and contrast sensitivity of 1.7 log CS was required. Participants mean visual acuity was −0.18 logMAR (*SD* = 0.06) and contrast sensitivity was 2.18 logCS (*SD* = 0.14). If hearing threshold levels exceeded 30 dB (average between 500 Hz and 1000 Hz), participants were excluded from the analysis (average of all frequencies: *M* = 23.1 dB, *SD* = 2.49 dB). The study was approved by the local ethics committee.

#### Materials

We used an interception setup similar to the ones reported in two recent studies by Schroeger et al. (2021) and Tolentino-Castro et al. (2021). Participants performed an interception task on a 43in. touchscreen (Iiyama PROLITE TF4338MSC-B1AG, 1,920 × 1,080, 60 Hz, 2.1 megapixel Full HD, Multi-Touch-Monitor). The experiment was programmed with PsychoPy 3 (Peirce et al., 2019), in the coder view using Python script.

**Fig. 1:**
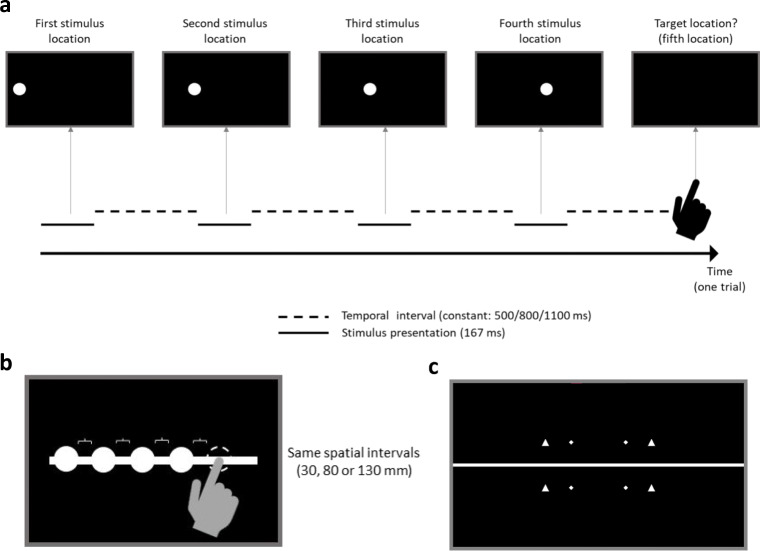
Experimental procedure. **a** After pressing the start button, the stimulus (ball) appeared four times at the screen, and the fifth location and time had to be anticipated. Each presentation of the stimulus was 167 ms and the temporal interstimulus intervals were constant (500, 800, or 1100 ms). **b** The spatial interstimulus intervals were constant, too (30, 80 or 130 mm). Please note that this is only an illustration, only one white circle was visible at a time. **c** Illustration of the reference objects presented in Experiment 2 to analyze gaze data

The visual stimuli were white circles (diameter 100 px) blurred with the help of Photoshop’s (Adobe Photoshop, EUA) Gaussian blur tool with radii of 0 (no blur) and 60 pixels. Stimuli were presented on a black background (similar to Sarrazin et al., 2004). In each trial a circle was presented four times successively for 167 ms each, with constant temporal and spatial interstimulus intervals between presentations. Temporal intervals were 500 ms, 800 ms, or 1100 ms and spatial interstimulus intervals 30 mm, 80 mm, or 130 mm (see Fig. 1a–b). Those values were chosen based on the properties of the touchscreen and pilot testing and are in the range of previously reported tau and kappa effects (e.g., temporal ISI: 250 ms–2,500 ms, spatial ISI: 30 mm and 50 mm in Abe, 1935). Piloting indicated that smaller temporal intervals made it impossible to reach the target location in time.

For the auditory stimuli 800 Hz pure tones were presented through two loudspeakers positioned at the right and left side of the touchscreen at the height of the ground line. Using the vector-based amplitude panning method (Pulkki, 1997) implemented in a MATLAB Script (Politis, 2016) the exact same temporal and spatial intervals between sound presentations and stimulus durations were produced as in the visual part. The virtual sound source is created through adjusting the signal amplitude of either of the two loudspeakers (intensity panning) based on the vectors between the listener, the loudspeakers, and the virtual sound source. Instead of blurring (visual part) for the auditory experiment two volumes (loudness) were used: ~55 dB and ~69 dB. The design was reduced to two levels of blur or volume based on pilot testing and to reduce the number of trials to a reasonable amount.

#### Procedure

Participants were seated in front of the touchscreen at approximately 50 cm (eyes to screen). That means that 1 cm on the screen (~20 px) refers to approximately 1.15° visual angle (but please note that participants were free to move/turn their heads). At the beginning participants took part in a familiarization phase of eight trials, using slightly different temporal (350 ms and 950 ms) and spatial intervals (10 mm and 100 mm) than in the main part of the experiments. During each practice trial, the white circle or sound (representing a ball) was presented on a white ground line successively four times (being occluded in between) before the fifth position had to be identified by tapping on the screen at the correct location and at the right time (see Fig. 1). Participants received feedback about both types of errors (spatial distance and temporal difference) during familiarization. Temporal and spatial intervals between stimuli were constant per trial but altered randomly between trials.

The main experiment consisted of six blocks of 36 randomized trials each. The main trials of the experiment were similar to those of the familiarization trials with one exception: exact feedback was not provided at the end of each trial. Instead, after each block a pause of at least 1 minute was included during which participants received feedback as a percentage score of the correctly hit trials. A hit was defined as tapping on the screen at a maximum horizontal distance of 73.5 mm from the correct location and a temporal deviation of not more than two times the stimulus presentation time (2 × 167 ms). These values were chosen based on pilot data with the aim to keep the participants sufficiently motivated. Visual or auditory stimuli were presented in two separate stimulus conditions and the order of conditions was counterbalanced across participants. Half of the participants started the experiment with the three visual blocks, whilst the other half first attended the three auditory blocks.

Combining all variables, the procedure of the main experiment resulted in 3 (temporal intervals) × 3 (spatial intervals) × 2 (blur levels/volumes) × 2 (condition: auditory vs. visual) = 36 conditions. Each combination was repeated 6 times, resulting in 216 trials. The experiment lasted about 1 hour (including pretests, instructions, experimental testing, and debriefing).

#### Data analysis

First, a difference score between the actual spatial interval and the spatial response and a difference score between the actual temporal interval and the temporal response were calculated. Based on these scores, for each participant outliers, defined as more than three interquartile ranges below or above the first or third quantile, were excluded. This resulted in 0.02–1.85% data exclusion in Experiments 1 and 2 (see Table S1 in the Online Supplement for further details). To evaluate the effect of the context variable on the primary task, linear mixed models were run, with either the spatial response or the temporal response as dependent variable (Schroeger et al., 2021; Tolentino-Castro et al., 2021). For both models the spatial interval, temporal interval, and blur/volume as well as their interactions were included as fixed and random effects for participants and random intercepts were modeled. Due to singularity and convergence problems the model was then reduced by excluding successively the random parts with the smallest variation (Barr et al., 2013; cf. Barr, 2013; Brauer & Curtin, 2018). As index of the tau effect the fixed effect of the temporal interval on the spatial response was evaluated, whereas the kappa effect was investigated by addressing the fixed effect of the spatial interval on the temporal response (each tested through model comparisons with and without the respective fixed effect). Blur or volume were included as additional predictors and the interaction between blur or volume and the context variable was regarded to evaluate whether the size of the relationship can be modulated by the informational value (i.e., representational noise). The standardized estimate (due to scaled data) of each effect will be reported and labeled as β.

### Results

#### Auditory condition

**Fig. 2:**
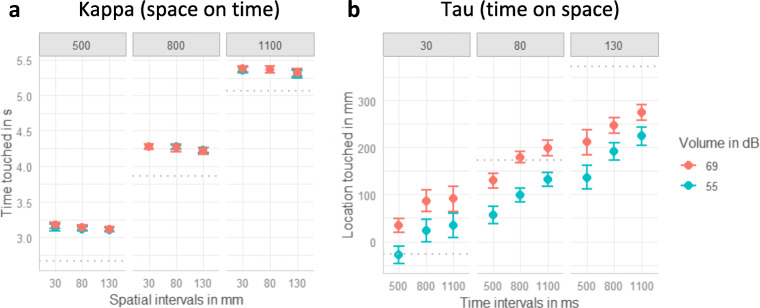
Plots of the auditory condition. Dots indicate means and error-bars indicate within-participant confidence intervals (Loftus & Masson, 1994). **a** Auditory kappa effect. Effect of volume, spatial and temporal intervals on the temporal response. One plot for each of the temporal intervals (500, 800, 1100 ms) is displayed. **b** Auditory tau effect. Effects of volume, spatial and temporal intervals on the spatial response (0 refers to the center of the screen and higher values indicate taps further to the right). One plot for each of the three spatial intervals (30 mm, 80 mm, 130 mm) is displayed. The gray dottet lines indicate the correct time (***a***) or location (***b***)

In the auditory condition, participants’ temporal response was significantly impacted by the temporal intervals, β = 0.90, χ^2^(1) = 221.86, *p* < .001, indicating that participants were sensitive to the temporal manipulation. Overall, participants reacted too late (see reaction times compared with dotted lines in Fig. 2a). As depicted in Fig. 2a, the longer the temporal intervals were (columns from left to right), the later participants touched the screen. There was a small but significant negative effect of spatial intervals, β = −0.02, χ^2^(1) = 6.64, *p* = .010, as depicted in Fig. 2a. For all three temporal intervals, the relationship between the spatial intervals and the temporal response tended to be slightly negative, as indicated by the negative slope. This contrasts with the expected positive impact of spatial interval on the temporal response and might indicate a reversed kappa effect. No other effects were significant (all *p*s > .471).

For the spatial response, the linear mixed model comparisons revealed a significant effect of spatial interval, β = 0.42, χ^2^(1) = 57.03, *p* < .001. The more distant the stimuli were (columns from left to right in Fig. 2b), the further to the right (in movement direction) participants tapped, confirming that participants were able to dissociate the varying spatial intervals. Likewise, louder sounds (red dots in Fig. 2b) led to spatial interception locations further to the right, β = 0.38, χ^2^(1) = 59.81, *p* < .001. In line with the hypothesis of an auditory tau effect, increasing temporal intervals resulted in reactions further to the right, β = 0.17, χ^2^(1) = 39.28, *p* < .001, as depicted by the positive slopes in the three columns of Fig. 2b. There was a nonsignificant trend for an interaction between spatial and temporal intervals, β = −0.02, χ^2^(1) = 2.98, *p* = .084, indicating that the effect of temporal intervals tended to increase with increasing spatial intervals. None of the other interactions were significant (all *p*s > .130).

#### Visual condition

**Fig. 3:**
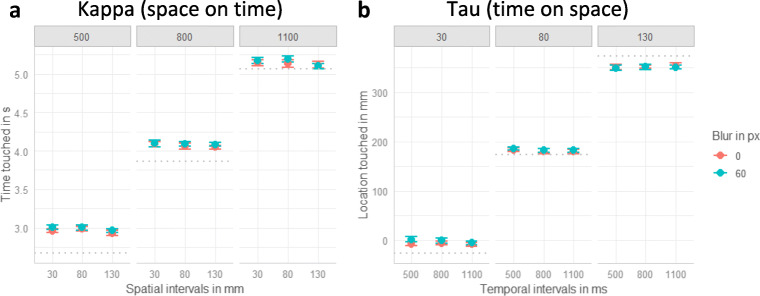
Plots of the visual condition. Dots indicate means and error-bars indicate within-participant confidence intervals (Loftus & Masson, 1994). **a** Visual kappa effect. Effect of blur, spatial and temporal intervals on the temporal response. One plot for each of the temporal intervals (500, 800, 1100 ms) is displayed. **b** Visual tau effect. Effects of blur, spatial and temporal intervals on the spatial response (0 refers to the center of the screen and higher values indicate taps further to the right). One plot for each of the three spatial intervals (30 mm, 80 mm, 130 mm) is displayed. The gray dottet lines indicate the correct time (***a***) or location (***b***)

The analysis of the temporal response in the visual data revealed that, overall, participants reacted too late, as can be seen in Fig. 3a (dotted line indicates the correct time and participants mostly reacted later). Approving the manipulation check, participants tapped the screen later with increasing temporal interval, β = 0.95, χ^2^(1) = 247.95, *p* < .001 (see three columns of Fig. 3a). Additionally, when stimuli were blurred (blue dots in Fig. 3a) participants tended to react later, but there was only a small effect, β = 0.03, χ^2^(1) = 7.74, *p* = .005. There was a negative effect of spatial intervals on the temporal response, β = −0.02, χ^2^(1) = 4.09, *p* = .043. As depicted by the slightly negative slope for each column in Fig. 3a, participants touched the screen earlier with increasing spatial intervals, again suggesting a reversed kappa effect. None of the interactions between the three predictors reached significance (all *p*s > .324).

The spatial response to visually presented stimuli was significantly impacted by the spatial intervals, β = 0.97, χ^2^(1) = 243.62 *p* <.001 (manipulation check). The longer the spatial intervals were (see three columns from left to right in Figure 3b), the further to the right participants touched the screen. There was a small but significant effect of blur, β = 0.01, χ^2^(1) = 4.72, *p* = .030, indicating that participants touched the screen slightly more to the right for blurred stimuli (blue dots in Fig. 3 b). Most importantly there was no significant effect of the temporal intervals (*p* > .136), indicating no visual tau effect (all three slopes in Fig. 3b are close to zero). The two-way interactions between spatial intervals and temporal intervals, β = 0.01, χ^2^(1) = 5.30, *p* = .021, and between spatial intervals and blur level reached significance, β = −0.02, χ^2^(1) = 13.00, *p* < .001. All other interactions did not reach significance (all *p*s > .232).

### Discussion

Here, we tested whether spatiotemporal illusions like *tau* and *kappa* effects would impact motor responses, specifically, in a manual interception task. Results support the suggested *tau* effect, that is, the effect of temporal intervals on spatial responses for auditory stimuli. This is in line with previous research reporting, for instance, a *tau* effect for auditory stimuli on relative judgements (e.g., Jones & Huang, 1982) and in a memory task (Sarrazin et al., 2007). In contrast to our predictions, for visual stimuli the interception timing was not delayed with increasing spatial intervals. In fact, quite an opposite pattern of results was observed. That is, there was even a small effect in the opposite direction, potentially pointing to a *reversed kappa* effect that was present for auditory stimuli, too. A negative effect of spatial intervals on the temporal response, however, is in line with results reported by Roy et al. (2011) in an auditory classification task (i.e., whether the presented sound was a long or short sound). The authors explained this finding with the internal clock model of time perception (Treisman et al., 1990). According to this model time perception functions through a so-called pacemaker which is emitting pulses. These pulses are then recorded and accumulated by another unit in the system. With increasing distance between two stimuli, more attention is shifted toward localizing those stimuli and therefore less attentional resources remain on the temporal task. Consequently, pulses are missed resulting in a smaller total number of accumulated pulses. In the end, participants perceive a shorter temporal interval because less pulses were counted. Potentially, this phenomenon might explain the current results. However, as both the visual and auditory *reversed kappa* effects were very small and just reached significance, these results should be interpreted with caution.

We can think of three more possible explanations for the unexpected absent *classical kappa* effect: First, this is not the first study finding no evidence for a transfer of visual illusions to actions. Previous research on action tasks, namely interception and grasping, provided mixed results: many studies report a transfer of illusion effects (de la Malla et al., 2019; de la Malla et al., 2018; Franz et al., 2000; Medendorp et al., 2018), others find no such effects (e.g., Aglioti et al., 1995; Haffenden & Goodale, 1998). A study on throwing performance reported mixed findings (Cañal-Bruland et al., 2013). We argue that the current results might therefore add to the ongoing debate about different visual processing streams for perception versus action (Goodale & Milner, 1992; Goodale et al., 1991), but it should be noted that other reasons for the missing effects are possible. Second, participants might know about their bias and by controlling for it, they might overcorrect, thereby nullifying (or even reversing) the expected effect. Third, as previous research suggests, the difficulty of the task is an important prerequisite for the illusions (cf. Jones & Huang, 1982). Tasks in which the primary judgement was relatively easy, revealed reduced or even no effects (Jones & Huang, 1982): for instance, musicians showed no auditory *tau* effect in a task where the primary judgement was about frequencies (cf. Jones & Huang, 1982); *tau* and *kappa* in a memory task were only found for varying compared with constant spatial and temporal intervals (Sarrazin et al., 2004; Sarrazin et al., 2007); and the *tau* effect decreases with decreasing signal duration supposedly due to worse spatial representations for short presentation times (Bill & Teft, 1972). This latter argument can be explained by the representational noise hypothesis introduced before (Cai & Wang, 2021). The noisier a representation is, the more prone to influences it will be. Assuming that the amount of noise corresponds to task difficulty, an easy task for the primary judgement would result in a reduced or absent impact of the context. If this was the case in the current visual condition, this would suggest that the temporal task was relatively easy. Post-hoc analysis providing initial evidence for this argument are reported in the Online Supplement (Fig. S1). This idea of representational noise is also in line with previous accounts on accuracy in interception suggesting that uncertainty in spatial localization might increase the reliance on prior information (Nelson et al., 2019). In our case, instead of priors, additionally available information (context) might impact performance. If indeed task difficulty in relation with representational noise can explain absent effects, it would be advantageous to include a measure of task difficulty in future analyses. Given that originally *tau* and *kappa* were found for fewer presentations of spatial and temporal intervals (typically one or two) and that not all effects were present in the current task with repeated presentation, it is arguable that repetition may have decreased the task difficulty resulting in absent or small effects. If the number of repetitions (“events”) makes the task easier by providing more time and presentations to learn and potentially adjust one’s predictions, a measure of difficulty might be included when having access to participants’ predictions on earlier stimulus events within each trial. A growing body of research shows that eye tracking might represent such a time-series-measure appropriate to evaluate motion prediction in interception tasks (for an overview, see Fooken et al., 2021). Eye movements may hence provide insights and help validate the new paradigm as a sensitive measure of perceptual biases, thereby indicating whether the dissociation between perception and action may account for the unexpected effects.

To summarize, whilst the auditory tau effect supports the initial hypothesis and is in line with previous research, the absence or even reversed visual kappa effect contrasts with most of previous reports. To (i) replicate the interception results and (ii) address two possible explanations for the absent *typical kappa* effects, a second experiment including eye-tracking measurements was conducted.

## EXPERIMENT 2

The aim of Exp. 2 was to test whether the gap between perception and action explains why increasing spatial intervals did not increase the temporal intervals and to identify the role of stimulus repetition (“events”) on motion prediction. Therefore, we replicated Exp. 1 while additionally measuring eye movements.

### Methods

#### Participants

In total 40 participants (19 male, *M*_Age_ = 24.2 years, *SD*_Age_ = 3.3 years; sample size similar to previous studies on interception, e.g., Schroeger et al., 2021) who did not enroll in Experiment 1 took part in the second experiment. Of the initially 45 collected data sets, five were excluded from the analysis because participants did not fulfil the vision requirements (3) or due to technical problems with the eye-tracking measurement (2). All requirements were identical to Experiment 1. The eye-tracking data of eight participants could not be analyzed due to one of the following issues: extreme head rotation (*n* = 1), interference of clothes or accessories with the automated analysis algorithm (*n* = 2), reference objects (see Fig. 1c) were partially cut, completely out of frame or occluded by participants’ hands (*n* = 5). This means that finally gaze data of 32 participants entered the analysis. For detailed descriptive statistics see Table 1.

**Table 1:** Descriptive statistics about the participants of Experiment 2

	Interception data (*N* = 40)		Gaze data (*N* = 32)	
Variable	*M*	*SD*	*M*	*SD*
Age (years)	22.78	2.58	22.56	2.37
Visual acuity (logMAR)	−0.14	0.08	−0.13	0.09
Contrast sensitivity (logCS)	2.15	0.16	2.14	0.16
Hearing threshold (dB)	23.54	1.95	23.81	1.99

#### Materials and procedure

Materials and Procedure were identical to Experiment 1 with one exception: Due to an automatic analysis algorithm based on visual object detection using OpenCV (Bradski, 2000) for the eye tracking data (see below), eight reference objects (visual objects: four triangles and four rectangles) were presented on the screen within each trial (see Fig. 1c). Participants were informed about these reference objects, and it was explained that they were only used for technical reasons and not important for the task.

#### Eye tracking

To record eye-tracking data, the portable system SMI ETG-2.6-1648-844 (SensoMotoric Instruments, Teltow, Germany; sampling frequency: 120 Hz for each eye, 30 Hz front camera) was used. Scan path videos were exported via the SMI BeGaze software and then analyzed frame by frame in Python (van Rossum & Drake Jr, 1995) with a self-written script using Spyder (Raybaut, 2009), Open CV (Bradski, 2000), math (van Rossum, 2020), matplotlib (Hunter, 2007), numpy (Harris et al., 2020), and pandas (McKinney, 2010). To do so, each frame recorded in reference to the viewer was transformed in reference to the screen (for a similar implementation, see MacInnes et al., 2018) and the gaze location was extracted through object detection. The code can be retrieved from the OSF (https://osf.io/9nx3u/). The gaze locations (*x* and *y* coordinates on the touchscreen) per frame were saved and then analyzed in R, using the package “saccades” (von der Malsburg, 2015) to categorize fixations and saccades, and the packages afex (Singmann et al., 2021), dplyr (Wickham et al., 2018), ggplot2 (Wickham, 2009), lmerTest (Kuznetsova et al., 2017), openxlsx (Schauberger & Walker, 2021), and reshape (Wickham, 2007) for the statistical analysis.

In contrast to the manual interception data, for the gaze data, earlier gaze locations and reaction times to the stimuli were used in the linear mixed models. Data regarding the third, fourth, and fifth event (appearance of the ball) were considered and included as another factor (“event”). The first and second event were excluded because they were needed to build the first spatial and temporal interval meaning that no prediction is possible at that time of the trial. As relevant measures, the final fixation before the target appeared at Event 3, 4, or (predicted) 5, was analyzed because previous studies showed that participants tend to fixate, for instance, predicted target locations in advance (Land & McLeod, 2000; Mann et al., 2019). Therefore, the temporal dependent variable was defined as the start of the final fixation before the target appeared, and the location where participants fixated immediately before the following event was taken as the spatial dependent variable. Additionally, the gaze location at the moment of interception was analyzed and these results are reported in the Online Supplement. Effects of and interactions between temporal intervals, spatial intervals, event, and volume (in dB) or blur were modeled.

### Results

#### Interception performance

**Fig. 4:**
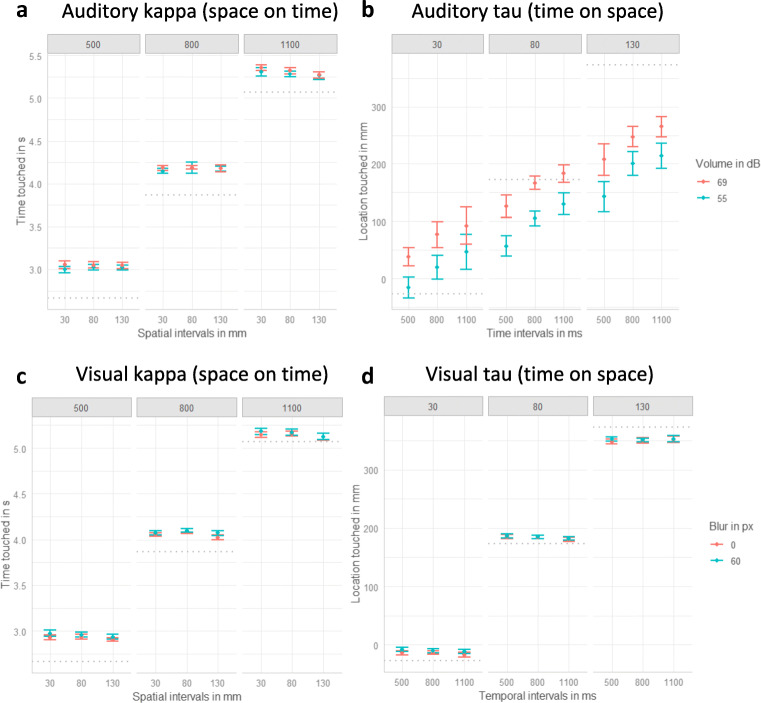
Interception results of Experiment 2. Dots indicate means and error-bars indicate within-participant confidence intervals (Loftus & Masson, 1994). **a** Auditory kappa effect. Effect of volume, spatial and temporal intervals on the temporal response. One plot for each of the temporal intervals (500, 800, 1100 ms) is displayed. **b** Auditory tau effect. Effects of volume, spatial and temporal intervals on the spatial response (0 refers to the center of the screen and higher values indicate taps further to the right). One plot for each of the three spatial intervals (30 mm, 80 mm, 130 mm ) is displayed. **c** Visual kappa effect. Effect of blur, spatial and temporal intervals on the temporal response. One plot for each of the temporal intervals (500, 800, 1100 ms) is displayed. **d** Visual tau effect. Effects of blur, spatial and temporal intervals on the spatial response (0 refers to the center of the screen and higher values indicate taps further to the right). One plot for each of the three spatial intervals (30 mm, 80 mm, 130 mm) is displayed. The gray dottet lines indicate the correct time (**a**, **c**) or location (**b**, **d**).

Overall, the results of the interception response of Experiment 2 replicated the results of Experiment 1: visually only a small, reversed kappa effect, β = −0.01, χ^2^(1) = 9.05, *p* < .003, but no significant tau effect was found, β = −0.01, χ^2^(1) = 2.38, *p* = .123; auditorily a significant tau, β = 0.16, χ^2^(1) = 23.57, *p* < .001, but no kappa effect was found, β = −0.01, χ^2^(1) = 1.30, *p* = .254 (for detailed results, see Table 2 and Fig. 4).

**Table 2:** Results of the linear mixed models’ analysis for the interception performance in Experiment 2

Auditory kappa (temporal response)	*df*	χ^2^	*p*	Auditory tau (spatial response)	*df*	χ^2^	*p*
spatial_ISI	1	1.30	.254	spatial_ISI	1	49.78 ***	<.001
temporal_ISI	1	228.30 ***	<.001	temporal_ISI	1	23.57 ***	<.001
blur	1	7.59 **	.006	volume	1	48.91 ***	<.001
spatial_ISI: temporal_ISI	1	9.39 **	.002	spatial_ISI: temporal_ISI	1	0.46	.498
spatial_ISI:blur	1	3.32 +	.069	spatial_ISI:volume	1	0.08	.772
temporal_ISI:blur	1	0.13	.719	temporal_ISI:volume	1	2.98 +	.084
spatial_ISI: temporal_ISI:blur	1	0.03	.868	spatial_ISI: temporal_ISI:volume	1	0.12	.730
Visual kappa (temporal response)	df	χ^2^	*p*	Visual tau (spatial response)	df	χ^2^	*p*
spatial_ISI	1	9.05 **	.003	spatial_ISI	1	253.29 ***	<.001
temporal_ISI	1	245.72 ***	<.001	temporal_ISI	1	2.38	.123
blur	1	17.75 ***	<.001	volume	1	9.03 **	.003
spatial_ISI: temporal_ISI	1	0.25	.615	spatial_ISI: temporal_ISI	1	5.40 *	.020
spatial_ISI:blur	1	0.18	.670	spatial_ISI:volume	1	2.40	.121
temporal_ISI:blur	1	1.01	.315	temporal_ISI:volume	1	0.51	.473
spatial_ISI: temporal_ISI: blur	1	0.24	.626	spatial_ISI: temporal_ISI:volume	1	0.25	.618

#### Gaze behavior

##### Auditory condition

**Fig. 5:**
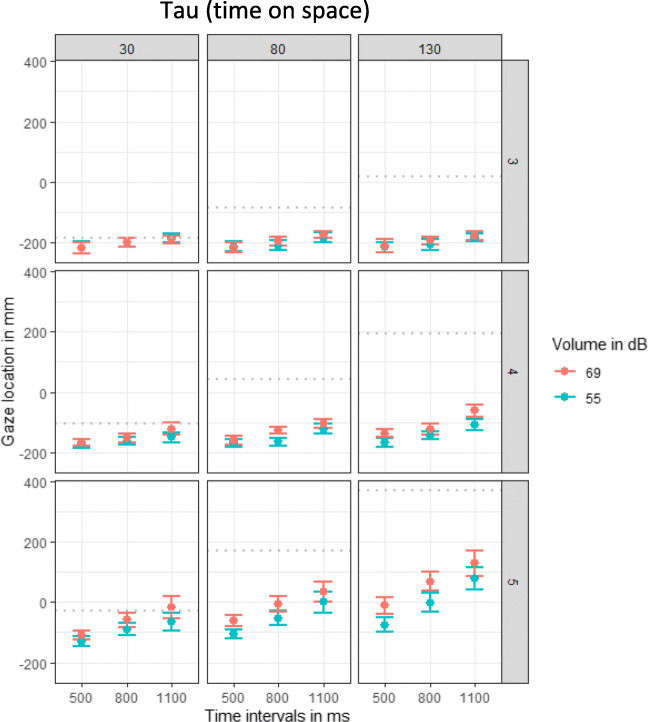
Plots of the auditory tau effect per event (third, fourth, or fifth apearance of the ball). Effect of volume, spatial and temporal intervals on the location of the final fixation. One plot for each of the temporal intervals (500, 800, 1100 ms) is displayed. Dots indicate means and error bars indicate within-participant confidence intervals. The gray dottet lines indicate the correct location

The location of the final fixation before the sound was started (or before the participant intercepted) was analyzed to evaluate a possible tau effect. The linear mixed model comparisons revealed a significant effect of spatial intervals, β = 0.17, χ^2^(1) = 35.73, *p* < .001, and volume (dB), β = 0.22, χ^2^(1) = 33.80, *p* < .001. With increasing spatial intervals and for louder sounds, participants fixated further to the right (see Fig. 5: the fixation timing increases for columns from left to right and for the red condition compared with the blue condition). Most importantly, with increasing temporal intervals participants fixated more to the right, β = 0.23, χ^2^(1) = 35.81, *p* < .001. Furthermore, the event revealed a significant effect, β = 0.61, χ^2^(1) = 59.42, *p* < .001. There were significant interactions between the spatial and temporal intervals β = 0.05, χ^2^(1) = 14.37, *p* < .001, the spatial intervals and event, β = 0.15, χ^2^(1) = 33.06, *p* < .001, the temporal intervals and event, β = 0.11, χ^2^(1) = 23.66, *p* < .001, volume and event, β = 0.15, χ^2^(1) = 29.84, *p* < .001, and spatial intervals, temporal intervals and event, β = 0.04, χ^2^(1) = 19.78, *p* < .001. There was a non-significant trend for an interaction between temporal intervals and volume, β = 0.03, χ^2^(1) = 3.07, *p* = .080. No other effects reached significance (all *p*s > .086).

**Fig. 6:**
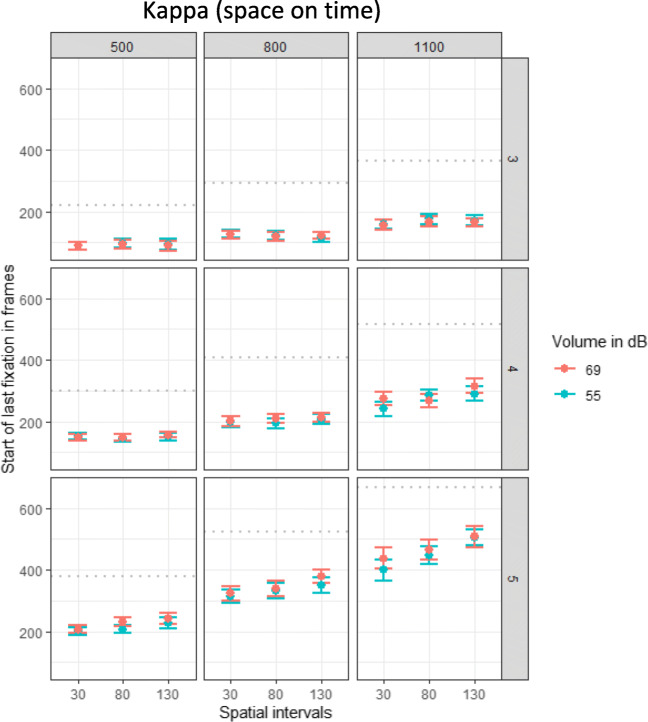
Plots of the auditory kappa effect per event (third, fourth, or fifth apearance of the ball). Effect of volume, spatial and temporal intervals on the start of the final fixation (in frames). Data were recorded with 120 frames per second. One plot for each of the temporal intervals (500, 800, 1100 ms) is displayed. Dots indicate means and error bars indicate within-participant confidence intervals. The gray dottet lines indicate the correct time

The analysis of the timing of the final fixation revealed significant effects of temporal intervals, β = 0.45, χ^2^(1) = 87.71, *p* < .001, volume, β = 0.06, χ^2^(1) = 5.40, *p* = .020, events, β = 0.64, χ^2^(1) = 80.87, *p* < .001, and most importantly, spatial intervals, β = 0.08, χ^2^(1) = 26.19, *p* < .001. Logically, the longer the temporal intervals were (see Fig. 6 three columns from left to right) or the later the ball event was (see Fig. 6 three rows top-down) the later participants started their final fixation. Additionally, the larger the spatial interval were, the later the final fixation was initiated, as can be seen by the positive slope in each grid of Fig. 6. The interaction between spatial and temporal intervals, β = 0.04, χ^2^(1) = 15.53, *p* < .001, spatial intervals and event, β = 0.07, χ^2^(1) = 23.80, *p* < .001, temporal intervals and event, β = 0.21, χ^2^(1) = 68.22, *p* < .001, and volume and event, β = 0.06, χ^2^(1) = 10.09, *p* = .001, reached significance. With increasing stimulus repetition (event), the effect of spatial intervals on the timing of the last fixation increased, as indicated by the increasing positive slope from top to down). Additionally, there was a significant three-way interaction between spatial intervals, temporal intervals, and event, β = 0.02, χ^2^(1) = 10.18, *p* = .001. All other interactions did not reach significance (all *p*s > .175) For a visualization of the results, see Fig. 6.

##### Visual condition

**Fig. 7:**
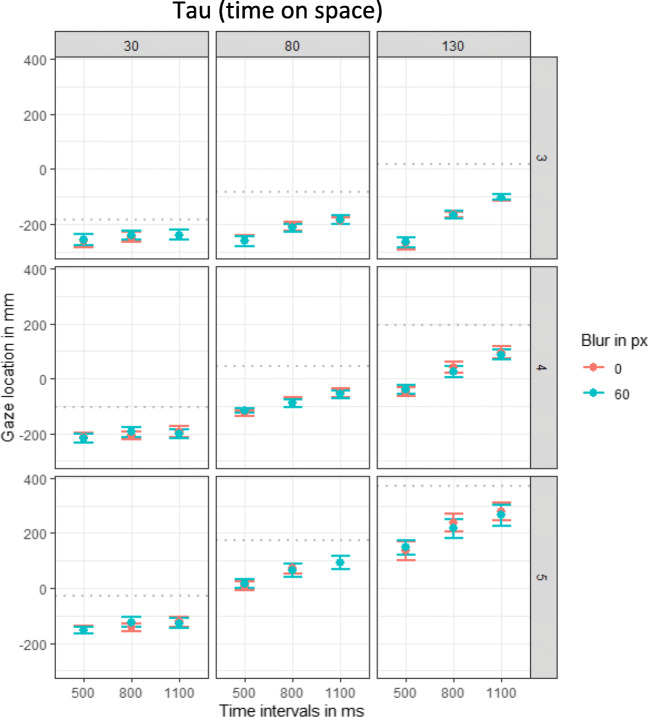
Plots of the visual tau effect per event (third, fourth, or fifth apearance of the ball). Effect of blur, spatial and temporal intervals on the location of the final fixation. One plot for each of the temporal intervals (500, 800, 1100 ms) is displayed. Dots indicate means and error bars indicate within-participant confidence intervals. The gray dottet lines indicate the correct location

To analyze the tau effect in the gaze data, the location of the final fixation before the ball appeared (or before the participant intercepted) was examined. Results of the visual data revealed that the spatial interval predicted where participants fixated, β = 0.52, χ^2^(1) = 74.53, *p* < .001, and the temporal intervals impacted the gaze location, β = 0.20, χ^2^(1) = 74.93, *p* < .001. Additionally, there was a significant effect of event, β = 0.63, χ^2^(1) = 86.89, *p* < .001, and an interaction between temporal and spatial intervals, β = 0.12, χ^2^(1) = 75.79, *p* < .001, indicating that the effect of the spatial intervals on the gaze location was larger for longer temporal intervals. There was also a significant interaction between spatial intervals and event, β = 0.28, χ^2^(1) = 83.20, *p* < .001, and a significant three-way interaction between spatial intervals, temporal intervals and event, β = −0.02, χ^2^(1) = 10.82, *p* = .001. All other effects did not significantly affect the gaze location of the final fixation (all *p*s > .171). These effects are depicted in Fig. 7.

**Fig. 8:**
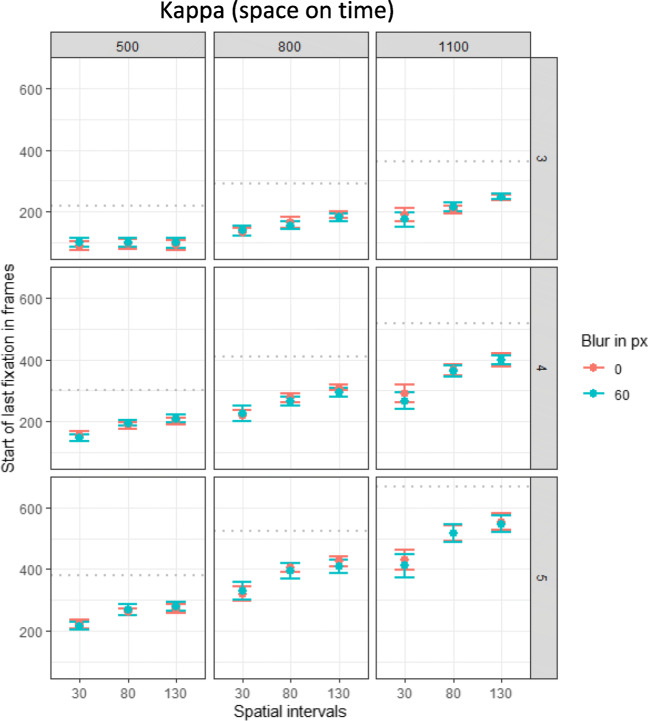
Plots of the visual kappa effect per event (thrid, fourth or fifth appearance of the ball). Effect of blur, spatial and temporal intervals on the start of the final fixation (in frames). Data were recorded with 120 frames per second. Dots indicate means and error bars indicate within-participant confidence intervals. One plot for each of the temporal intervals (500, 800, 1100 ms) is displayed. The gray dottet lines indicate the correct time

As indicator of a kappa effect in the gaze data, the time participants started their final fixation was analyzed. The analysis revealed a significant positive effect of the temporal intervals on the timing of the last fixation, β = 0.49, χ^2^(1) = 91.76, *p* < .001. As can be seen in Fig. 8 in the three columns from left to right, the last fixation was initiated later with increasing temporal intervals. Most importantly, there was a positive relation between the spatial intervals and the start of the final fixation, β = 0.20, χ^2^(1) =31.78, *p* < 001. These two effects were further explained by their significant two-way interaction, β = 0.08, χ^2^(1) = 27.82, *p* < .001, indicating that the positive relation between spatial intervals and timing of fixation increased with increasing temporal intervals (the positive slope increases from left to right in Fig. 8). Additionally, there was a significant effect of event, β = 0.62, χ^2^(1) = 94.73, *p* < .001, and significant interactions between spatial intervals and event, β = 0.06, χ^2^(1) = 17.22, *p* < .001, temporal intervals and event, β = 0.14, χ^2^(1) = 77.44, *p* < .001. The effect of the spatial intervals on the initiation of the final fixation increased with the number of target events (increasing slopes from top to down in Fig. 5). No other effects reached significance (all *p*s > .123).

### Discussion

In Experiment 2 we aimed to replicate the results found in Experiment 1, namely an auditory tau effect for interception performance, and to extend and explain these findings, especially the absent visual kappa effect, by using eye tracking measures. Regarding the interception response, overall, we successfully replicated the effects found in Experiment 1: an auditory tau effect, a small reversed visual kappa effect, and no visual tau effect. In contrast to Exp. 1, where a small, reversed kappa effect was evident for the auditory data as well, the results of Exp. 2 provide no significant effect. The gaze data revealed indications of visual and auditory tau and kappa effects. The longer the temporal intervals, the further participants moved their gaze for the final fixation before stimulus presentation (either visually or auditorily). Additionally, for both modalities, participants initiated their final fixation before presentation later, the larger the spatial interval were.

As eye movements have been found to be highly correlated with motion perception and prediction (Schütz et al., 2011), this might indicate that the adapted paradigm was able to produce spatiotemporal illusions, at least at the level of spatiotemporal perception and prediction. Interestingly, these effects did not transfer to interception performance: For both modalities, the spatial intervals impacted when participants fixated but revealed small, reversed effects for interception. Although participants’ gaze location was affected by the temporal intervals in the visual condition, they did not intercept at those fixation locations. Auditorily both, gaze and interception location depended on the temporal intervals. These results will be discussed in more detail in the following General Discussion.

## GENERAL DISCUSSION

Intercepting a moving object relies on predicting the object’s trajectory in space and time and executing precise movements (e.g., Fiehler et al., 2019; Land & McLeod, 2000). Interception performance might therefore be influenced by interrelations between spatial and temporal processing, as found for spatial and temporal judgements (e.g., Helson & King, 1931). A recent review suggests that seemingly contradictory hypotheses about spatiotemporal interrelations as proposed by ATOM versus CMT can be consolidated when including sensory modality as a moderating variable (Loeffler et al., 2018). Following this rationale, we proposed two hypotheses taking into account different sensitivities for spatial and temporal information across sensory modalities: (i) in an auditory condition, effects of temporal intervals on spatial interception responses were predicted (*tau* effect), whilst manipulations of spatial intervals were assumed to have only small or no impact on temporal responses (no or small *kappa* effect); (ii) for visual stimuli larger effects of spatial manipulations on temporal responses were expected (*kappa* effect), whereas temporal manipulations should not or only marginally impact spatial responses (no or small *tau* effect).

Our findings provided evidence for spatiotemporal interrelations in a new form of tasks—namely, (auditory) interception—as compared with the previously reported effects on relative judgments (e.g., Jones & Huang, 1982) and memory retrieval (Sarrazin et al., 2004). Moreover, the results indicate that modality plays an important role as concerns the contributions of spatial and temporal characteristics of a task (O’Connor & Hermelin, 1972; Recanzone, 2009; Schmiedchen et al., 2012). Both experiments showed that in the auditory condition interception performance revealed a significant *tau*, but no classical (yet in Exp. 1 a small and reversed) *kappa* effect. In contrast to our predictions, also for visual stimuli no classical, but again a small and surprisingly reversed *kappa* effect was found across experiments. Also, in both experiments, no visual tau effect was found, in line with our predictions. Given that there was an auditory but no visual *tau* effect, together these results seem to support the notion that sensory modality plays an important role and should be considered when investigating spatiotemporal interrelations in interception.

### Debate on ATOM versus CMT

The current results are adding to the debate on ATOM (Walsh, 2003) and CMT (Lakoff & Johnson, 1980). In contrast to previous research applying tau and kappa paradigms to solve the controversy between those theories (Reali et al., 2019), the current results clearly contradict the asymmetrical relationship proposed in CMT with higher impact of spatial characteristics on temporal judgements. Rather than finding a symmetrical or asymmetrical relationship between spatial and temporal representations, the size of effects in either direction may actually depend on other factors. Here, we showed that sensory modality is one of those factors. While previous research showed that for visual tasks typically larger effects of space on temporal judgements are found (e.g., Casasanto & Boroditsky, 2008), the current results revealed the opposite pattern for auditory stimuli. This seems to indicate that both the predictions of CMT of ATOM can be met depending on sensory modality. In this vein, perhaps the best way to capture and conceptualize the relationship between time and space is offered by the theory of representational noise (Cai & Wang, 2021).

### Noise and modality

Cai and Wang (2021) propose that the interrelations between spatial and temporal representations are affected by the amount of representational noise. Assuming different levels of noise under varying sensory conditions might therefore be the theoretical basis of the presented results. The amount of noise for each modality might be inferred from the sensitivity of the respective modality toward spatial versus temporal information. The finding that the auditory system appears to be dominated by temporal compared with spatial information (O’Connor & Hermelin, 1972; Recanzone, 2009) together with the imprecision of auditory localization in humans compared with visual localization (Middlebrooks & Green, 1991) points to the fact that less representational noise may be expected for temporal information. If spatial representations were very noisy, they may have been influenced by concurrent temporal information, thereby explaining why participants touched the screen further in movement direction of the stimulus.

Contrary to our hypothesis, blur and volume manipulations did not impact the size of the effects, questioning the assumption that they would increase representational noise of either spatial or temporal representations. Potentially these manipulations have not been appropriate for that purpose, especially as they mainly address the stimulus locations, but not directly the spatial and temporal intervals. For future research it would be beneficial to explicitly test the predicted changes in representational noise, before including them as manipulations on spatiotemporal interrelations. One problem with blurring stimuli is that an impact on (spatial localization) performance often is only found (if at all) for very high blur levels (Alais & Burr, 2004; Kramer et al., 2019; Mann et al., 2010). An alternative visual manipulation might hence need much higher levels of visual blur. For sounds, it is known that broadband noise can be much easier localized when compared with sinus sounds which might therefore be a better candidate as a potential manipulation of spatial representational noise for auditory stimuli. Our results of the auditory manipulation revealed only a main effect of volume on the interception location. Louder sounds were perceived to go further. Similar results of sound intensity on localization were obtained by Cañal-Bruland et al. (2018) for anticipation in tennis. Their investigation suggests that next to visual information obtained from a tennis stroke, auditory cues are used to estimate the ball’s trajectory. Louder sounds are associated with longer trajectories potentially because they are linked to stronger strokes. This is supported by the notion that auditory cues are more informative for shot power discrimination than visual cues (Sors et al., 2017), and that grunting intensities impact spatial predictions in tennis (Müller et al., 2019). Similar processes might have influenced participants’ interception in the current study, if louder sounds were associated with stronger bounces. However, this manipulation seems not to have increased noise for either the spatial or the temporal representation.

The idea, that spatiotemporal illusions depend on variability or uncertainty was also raised by Brooks et al. (2019) and shown in Schmiedchen et al. (2013) for other spatiotemporal interrelations. Brooks et al. (2019) argued that reducing information to fulfill the task, increases the effect of such illusions, as can also be explained by a Bayesian model (e.g., Goldreich, 2007; Goldreich & Tong, 2013). For future research, the use of Bayesian models might proof especially helpful to address the role of representational noise. In Bayesian cue integration models (for an overview, see Seilheimer et al., 2014), noise, operationalized as the reliability of the sensory input, accounts for the weighting and integration of signal from various sensory modalities. To explicitly address the effect of noise on the size of spatiotemporal biases, such models may be particularly helpful and insightful.

### Perceptual effects in interception

Interestingly, the absence of the illusion’s effect in interception is in contrast with previous research on the transfer of visual perceptual illusions to interception (e.g., de la Malla et al., 2019; de la Malla et al., 2018). Despite using a similar interception task, the current study differed in the type of stimuli applied to evoke an illusion: These previous interception studies investigated illusory motion, whereas the present stimuli might be rather comparable to, for instance, size illusions. Studies on such size illusions in motoric responses mostly applied grasping and throwing tasks. Overall, mixed results (Cañal-Bruland et al., 2013) have been reported with some studies providing evidence for a transfer (Franz et al., 2000; for a review see Medendorp et al., 2018) and others showing no such effects (Aglioti et al., 1995; Haffenden & Goodale, 1998).

In the following we argue that the missing effects in vision might not call for a general absence of such a transfer, but rather indicate the important role of additional factors. As alluded to above, the effects of space on time and vice versa seem to depend on the amount of representational noise. Asymmetrical effects of space on time are only expected when temporal noise is relatively high. If the temporal part of the task was simply too easy—meaning that participants were very certain/precise in their temporal response—no impact of spatial characteristics would be predicted. Further evidence for this notion was provided in the research on kappa and tau effects (e.g., Jones & Huang, 1982). For instance, longer stimulus presentation durations are associated with higher focus on spatial compared with temporal characteristics. That is, spatial characteristics are more precisely represented when each stimulus is presented for more time whereas temporal precision diminishes. Accordingly, Bill and Teft (1972) showed that the tau effect decreases with increasing signal duration. Additionally, Jones and Huang (1982) assumed that an increase of the entire duration of one trial makes it more difficult to remember the initial stimulus location. Therefore, the spatial interval should be less precisely represented. Consequently, they found that the tau effect increased, whilst the kappa effect decreased with increasing total time (Jones & Huang, 1982).

### Perceptual effects in eye movements

Interestingly, the gaze data of Exp. 2 largely deviate from the interception performance. Here, both effects were found for auditory and visual stimuli. Given that eye movements have been reported to be highly correlated with perceptual processes (Schütz et al., 2011), and tau and kappa have been reported for perceptual tasks, this finding might be interpreted as a first validation of the novel interception paradigm presented in this study to investigate these illusions.

Still, the discrepancy between interception and eye movement results are surprising given that eye movements were shown to contribute significantly to spatiotemporal prediction and temporal interception (Fooken et al., 2021). For instance, previous results indicate that fixation locations are highly correlated with interception locations (cf. Fooken et al., 2021). The divergent findings in the current study might underpin the suggested dissociation between perceptual (gaze) and action (interception) tasks, at least for visual information processing (Goodale & Milner, 1992; Goodale et al., 1991). Yet other explanations (e.g., task difficulty) cannot be ruled out. As concerns the role of task difficulty (cf. Huang & Jones, 1982), the number of repetitions of the target presentation and the ISI (events) did not decrease the effects. Quite the opposite, effects were largest for the last event, contradicting the idea that the task was too simple (low amount of representational noise) due to repeated presentation. Post hoc analyses (see Online Supplementary) rather showed that with increasing repetition the variability in the spatial response was increasing.

### Future perspectives

To the best of our knowledge, this study is the first to examine *tau* and *kappa* effects on interception performance. Therefore, the current study extends previous research in several ways regarding the application of the temporal and spatial task. While in early research on tau and kappa (e.g., Cohen et al., 1953; Helson & King, 1931), participants had to either focus on spatial or on temporal information (primary judgement) and ignore the second domain (context), here they had to process both information to successfully fulfil the task (to be in the right place at the right time). Moreover, compared with research on ATOM and CMT (e.g., Cai & Connell, 2015; Casasanto & Boroditsky, 2008), the current interception task differs as the dependent measure is an amalgam of spatial accuracy (being in the right place) and temporal accuracy (at the right time). Even if in some studies on ATOM and CMT participants were not informed prior to task execution about which information (spatial vs. temporal) they had to reproduce/judge until the stimulus presentation was finished (e.g., Casasanto & Boroditsky, 2008), this is the first study in which participants had to indicate both information in one spatiotemporal response (i.e., a single touch). This new method has certainly some advantages but also disadvantages. One the one hand, it is a step into more dynamic scenarios where the participant interacts with the environment, therefore strengthening ecological validity. On the other hand, it might have reduced the effects, if participants had divided their attention between both tasks with sometimes only focusing on the spatial and sometimes only focusing on the temporal demands. More robust effects might be expected, if participants would only focus on either the spatial or the temporal response. Future research with separate experiments for spatial vs. temporal prediction are needed to better understand those interrelations. Finally, daily life mostly confronts us with input from different modalities at the same time. To fully understand human processing of time and space, multisensory studies are needed. It was already shown that cross-modality tau (Kawabe et al., 2008) and kappa (Bausenhart & Quinn, 2018) effects can be observed when temporal information is presented auditorily and spatial information visually. Also, research in related areas, for instance, on the representational momentum (the final location of a disappearing moving object is perceived to be shifted in motion direction), indicates cross-modality effects from visually presented motion on tactile localization but not vice versa (Merz et al., 2020). Similarly, the research on *tau* and *kappa* in interception should be extended for different modalities providing either temporal or spatial or both information at the same time to fully understand whether and under which conditions such interrelations impact human behavior in real world behavior (i.e., outside the lab).

To summarize the current study adds to research on spatiotemporal interrelations by showing an auditory tau effect in manual interception, that is, an effect of temporal intervals between sounds on spatial interception performance. It provides initial empirical support for the role of sensory modality as a moderating factor consolidating seemingly contradictory predictions and findings of a theory of magnitude and the conceptual metaphor theory. The application of eye tracking further suggests differences in spatiotemporal interrelations between merely perceptual versus action tasks.

## Supplementary Information

ESM 1 (DOCX 308 kb)

## References

[CR1] Abe S (1935). Experimental study of the co-relation between time and space. Tohoku Psychologica Folia.

[CR2] Aglioti S, DeSouza JF, Goodale MA (1995). Size-contrast illusions deceive the eye but not the hand. Current Biology.

[CR3] Alais D, Burr D (2004). The ventriloquist effect results from near-optimal bimodal integration. Current Biology: CB.

[CR4] Alards-Tomalin D, Leboe-McGowan JP, Shaw JDM, Leboe-McGowan LC (2014). The effects of numerical magnitude, size, and color saturation on perceived interval duration. Journal of Experimental Psychology: Learning, Memory, and Cognition.

[CR5] Bach M (1996). The “Freiburg Visual Acuity Test”—Automatic measurement of visual acuity. Optometry and Vision Science.

[CR6] Bach M (2006). The Freiburg Visual Acuity Test-Variability unchanged by post-hoc re-analysis. Graefe’s Archive for Clinical and Experimental Ophthalmology.

[CR7] Barr DJ (2013). Random effects structure for testing interactions in linear mixed-effects models. Frontiers in Psychology.

[CR8] Barr, D. J., Levy, R., Scheepers, C., & Tily, H. J. (2013). Random effects structure for confirmatory hypothesis testing: Keep it maximal. *Journal of Memory and Language, 68*(3). 10.1016/j.jml.2012.11.001 10.1016/j.jml.2012.11.001 PMC3881361 24403724

[CR9] Bausenhart KM, Quinn KR (2018). On the interplay of visuospatial and audiotemporal dominance: Evidence from a multimodal kappa effect. Attention, Perception, & Psychophysics.

[CR10] Benussi, V. (1913). *Psychologie der Zeitauffassung* [Psychology of time perception] (Vol. 6). C. Winter.

[CR11] Bill JC, Teft LW (1972). Space-time relations: The effects of variations in stimulus and interstimulus interval duration on perceived visual extent. Acta Psychologica.

[CR12] Bradski G (2000). The OpenCV Library. Dr. Dobb’s Journal of Software Tools.

[CR13] Brauer M, Curtin JJ (2018). Linear mixed-effects models and the analysis of nonindependent data: A unified framework to analyze categorical and continuous independent variables that vary within-subjects and/or within-items. Psychological Methods.

[CR14] Brooks J, Seizova-Cajic T, Taylor JL (2019). Biases in tactile localization by pointing: Compression for weak stimuli and centering for distributions of stimuli. Journal of Neurophysiology.

[CR15] Cai ZG, Connell L (2015). Space-time interdependence: Evidence against asymmetric mapping between time and space. Cognition.

[CR16] Cai, Z. G., & Wang, R. (2021). Cross-dimensional magnitude interaction is modulated by representational noise: Evidence from space-time interaction. *Psychological Research. Advance online publication.*10.1007/s00426-020-01472-4 10.1007/s00426-020-01472-4 33580821

[CR17] Cañal-Bruland R, Müller F, Lach B, Spence C (2018). Auditory contributions to visual anticipation in tennis. Psychology of Sport and Exercise.

[CR18] Cañal-Bruland R, Voorwald F, Wielaard K, van der Kamp J (2013). Dissociations between vision for perception and vision for action depend on the relative availability of egocentric and allocentric information. Attention, Perception, & Psychophysics.

[CR19] Casasanto D, Boroditsky L (2008). Time in the mind: Using space to think about time. Cognition.

[CR20] Casasanto D, Fotakopoulou O, Boroditsky L (2010). Space and Time in the Child’s Mind: Evidence for a Cross-Dimensional Asymmetry. Cognitive Science.

[CR21] Cohen J, Hansel CEM, Sylvester JD (1953). A new phenomenon in time judgment. Nature.

[CR22] de la Malla C, Brenner E, de Haan EHF, Smeets JBJ (2019). A visual illusion that influences perception and action through the dorsal pathway. Communications Biology.

[CR23] de la Malla C, Smeets JBJ, Brenner E (2018). Errors in interception can be predicted from errors in perception. Cortex.

[CR24] Fiehler K, Brenner E, Spering M (2019). Prediction in goal-directed action. Journal of Vision.

[CR25] Fischman MG, Schneider T (1985). Skill level, vision, and proprioception in simple one-hand catching. Journal of Motor Behavior.

[CR26] Fooken J, Kreyenmeier P, Spering M (2021). The role of eye movements in manual interception: A mini-review. Vision Research.

[CR27] Fooken J, Spering M (2020). Eye movements as a readout of sensorimotor decision processes. Journal of Neurophysiology.

[CR28] Fooken J, Yeo S-H, Pai DK, Spering M (2016). Eye movement accuracy determines natural interception strategies. Journal of Vision.

[CR29] Franz VH, Gegenfurtner KR, Bülthoff HH, Fahle M (2000). Grasping visual illusions: No evidence for a dissociation between perception and action. Psychological Science.

[CR30] Gelb, A. (Ed.). (1914). Versuche auf dem Gebiete der Zeit–und Raumanschauung [Experiments in the field of time and space perception]. *Kongress fur Experimentelle Psychologie* [Congress of Experimental Psychology], 36–42.

[CR31] Goettker A, Braun DI, Schütz AC, Gegenfurtner KR (2018). Execution of saccadic eye movements affects speed perception. Proceedings of the National Academy of Sciences of the United States of America.

[CR32] Goettker A, Brenner E, Gegenfurtner KR, de la Malla C (2019). Corrective saccades influence velocity judgments and interception. Scientific Reports.

[CR33] Goldreich D (2007). A Bayesian perceptual model replicates the cutaneous rabbit and other tactile spatiotemporal illusions. PLOS ONE.

[CR34] Goldreich D, Tong J (2013). Prediction, postdiction, and perceptual length contraction: A Bayesian low-speed prior captures the cutaneous rabbit and related illusions. Frontiers in Psychology.

[CR35] Goodale MA, Milner AD (1992). Separate visual pathways for perception and action. Trends in Neurosciences.

[CR36] Goodale MA, Milner AD, Jakobson LS, Carey DP (1991). A neurological dissociation between perceiving objects and grasping them. Nature.

[CR37] Haffenden AM, Goodale MA (1998). The effect of pictorial illusion on prehension and perception. *The Brain & Neural*. Networks.

[CR38] Harris CR, Millman KJ, van der Walt SJ, Gommers R, Virtanen P, Cournapeau D, Wieser E, Taylor J, Berg S, Smith NJ, Kern R, Picus M, Hoyer S, van Kerkwijk MH, Brett M, Haldane A, Del Río JF, Wiebe M, Peterson P, Oliphant TE (2020). Array programming with NumPy. Nature.

[CR39] Helson H, King SM (1931). The tau effect: an example of psychological relativity. Journal of Experimental Psychology.

[CR40] Hodges NJ, Wyder-Hodge PA, Hetherington S, Baker J, Besler Z, Spering M (2021). Topical review: Perceptual-cognitive skills, methods, and skill-based comparisons in interceptive sports. Optometry and Vision Science : Official Publication of the American Academy of Optometry.

[CR41] Huang YL, Jones B (1982). On the interdependence of temporal and spatial judgments. Perception & Psychophysics.

[CR42] Hunter JD (2007). Matplotlib: A 2D graphics environment. Computing in Science & Engineering.

[CR43] Jones B, Huang YL (1982). Space-time dependencies in psychophysical judgment of extent and duration: Algebraic models of the tau and kappa effects. Psychological Bulletin.

[CR44] Kawabe T, Miura K, Yamada Y (2008). Audiovisual tau effect. Acta Psychologica.

[CR45] Kramer A, Röder B, Bruns P (2019). Feedback modulates audio-visual spatial recalibration. Frontiers in Integrative Neuroscience.

[CR46] Kuznetsova A, Brockhoff PB, Christensen RHB (2017). lmerTest package: Tests in Linear mixed effects models. Journal of Statistical Software.

[CR47] Lakoff G, Johnson M (1980). The Metaphorical Structure of the Human Conceptual System. Cognitive Science.

[CR48] Land MF, McLeod P (2000). From eye movements to actions: How batsmen hit the ball. Nature Neuroscience.

[CR49] Loeffler J, Cañal-Bruland R, Schroeger A, Tolentino-Castro JW, Raab M (2018). Interrelations between temporal and spatial cognition: The role of modality-specific processing. Frontiers in Psychology.

[CR50] Loffing F, Cañal-Bruland R (2017). Anticipation in sport. Current Opinion in Psychology.

[CR51] Loftus, G. R., & Masson, M. E. (1994). Using confidence intervals in within-subject designs. *Psychonomic Bulletin & Review, 1*(4), 476–490. 10.3758/BF03210951 10.3758/BF03210951 24203555

[CR52] MacInnes, J. J., Iqbal, S., Pearson, J., & Johnson, E. N. (2018). *Wearable Eye-Tracking for Research: Automated Dynamic Gaze Mapping and Accuracy/Precision Comparisons Across Devices.*10.1101/299925

[CR53] Mann DL, Abernethy B, Farrow D (2010). The resilience of natural interceptive actions to refractive blur. Human Movement Science.

[CR54] Mann DL, Nakamoto H, Logt N, Sikkink L, Brenner E (2019). Predictive eye movements when hitting a bouncing ball. Journal of Vision.

[CR55] McBeath MK (1990). The rising fastball: Baseball’s impossible pitch. Perception.

[CR56] McKinney WAO (2010). Data structures for statistical computing in python. Proceedings of the 9th Python in Science Conference.

[CR57] Medendorp WP, de Brouwer AJ, Smeets JBJ (2018). Dynamic representations of visual space for perception and action. Cortex.

[CR58] Merz S, Meyerhoff HS, Frings C, Spence C (2020). Representational momentum in vision and touch: Visual motion information biases tactile spatial localization. Attention, Perception, & Psychophysics.

[CR59] Middlebrooks JC, Green DM (1991). Sound localization by human listeners. Annual Review of Psychology.

[CR60] Müller F, Jauernig L, Cañal-Bruland R (2019). The sound of speed: How grunting affects opponents’ anticipation in tennis. PLOS ONE.

[CR61] Nelson JS, Baud-Bovy G, Smeets JBJ, Brenner E (2019). Accuracy of intercepting moving tactile targets. Perception.

[CR62] O’Connor N, Hermelin B (1972). Seeing and hearing and space and time. Perception & Psychophysics.

[CR63] Oudejans RRD, Michaels CF, Bakker FC, Dolné MA (1996). The relevance of action in perceiving affordances: Perception of catchableness of fly balls. Journal of Experimental Psychology: Human Perception and Performance.

[CR64] Peirce J, Gray JR, Simpson S, MacAskill M, Höchenberger R, Sogo H, Kastman E, Lindeløv JK (2019). PsychoPy2: Experiments in behavior made easy. Behavior Research Methods.

[CR65] Price-Williams DR (1954). The kappa effect. Nature.

[CR66] Politis, A. (2016). Microphone array processing for parametric spatial audio techniques [Doctoral Dissertation]. Aalto University.

[CR67] Pulkki, V. (1997). Virtual sound source positioning using vector base amplitude panning. *Journal of the Audio Engineering Society*, (45:6), 456–466.

[CR68] Raybaut, P. (2009). *Spyder-documentation.* Pythonhosted.org.

[CR69] Reali F, Lleras M, Alviar C (2019). Asymmetrical time and space interference in tau and kappa effects. Cogent Psychology.

[CR70] Recanzone GH (2009). Interactions of auditory and visual stimuli in space and time. Hearing Research.

[CR71] Roy, M., Kuroda, T., & Grondi, S. (2011). Effect of space on auditory temporal processing with a single-stimulus method. In P. Strumillo (Ed.), *Advances in sound localization.* InTech. 10.5772/14436

[CR72] Sarrazin J-C, Giraudo M-D, Pailhous J, Bootsma RJ (2004). Dynamics of balancing space and time in memory: Tau and kappa effects revisited. Journal of Experimental Psychology: Human Perception and Performance.

[CR73] Sarrazin J-C, Giraudo M-D, Pittenger JB (2007). Tau and Kappa effects in physical space: The case of audition. Psychological Research.

[CR74] Savelsbergh GJ, Whiting HT (1988). The effect of skill level, external frame of reference and environmental changes on one-handed catching. Ergonomics.

[CR75] Schauberger, P., & Walker, A. (2021). openxlsx: Read, write and edit xlsx files (R package Version 4.2.4) [Computer software]. https://CRAN.R-project.org/package=openxlsx

[CR76] Schmiedchen K, Freigang C, Nitsche I, Rübsamen R (2012). Crossmodal interactions and multisensory integration in the perception of audio-visual motion—A free-field study. Brain Research.

[CR77] Schmiedchen K, Freigang C, Rübsamen R, Richter N (2013). A comparison of visual and auditory representational momentum in spatial tasks. Attention, Perception, & Psychophysics.

[CR78] Scholz W (1924). Experimentelle Untersuchungen über die phänomenale Größe von Raumstrecken, die durch Sukzessiv-Darbietung zweier Reize begrenzt warden [Experimental investigations into the phenomenal size of spatial stretches that are delimited by the successive presentation of two stimuli]. Psychologische Forschung.

[CR79] Schroeger, A., Tolentino-Castro, J. W., Raab, M., & Cañal-Bruland, R. (2021). Effects of visual blur and contrast on spatial and temporal precision in manual interception. *Experimental Brain Research, 239*(11), 3343–3358. 10.1007/s00221-021-06184-8 10.1007/s00221-021-06184-8 PMC8542000 34480594

[CR80] Schütz, A. C., Braun, D. I., & Gegenfurtner, K. R. (2011). Eye movements and perception: A selective review. *Journal of Vision, 11*(5). 10.1167/11.5.9 10.1167/11.5.9 21917784

[CR81] Seilheimer RL, Rosenberg A, Angelaki DE (2014). Models and processes of multisensory cue combination. Current Opinion in Neurobiology.

[CR82] Singmann, H., Bolker, B., Westfall, J., Aust, F., & Ben-Sachar, M. S. (2021). afex: Analysis of factorial experiments (R package Version 1.0-1) [Computer software]. https://CRAN.R-project.org/package=afex

[CR83] Sors F, Murgia M, Santoro I, Prpic V, Galmonte A, Agostini T (2017). The contribution of early auditory and visual information to the discrimination of shot power in ball sports. of Sport and Exercise.

[CR84] Spering M, Schütz AC, Braun DI, Gegenfurtner KR (2011). Keep your eyes on the ball: Smooth pursuit eye movements enhance prediction of visual motion. Journal of Neurophysiology.

[CR85] Tolentino-Castro, J. W., Schroeger, A., Cañal-Bruland, R., & Raab, M. (2021). The impact of pitch on tempo-spatial accuracy and precision in intercepting a virtually moving ball. *Journal of Motor Behavior, 1–15*. 10.1080/00222895.2021.1933886 10.1080/00222895.2021.1933886 34180782

[CR86] Treisman M, Faulkner A, Naish PL, Brogan D (1990). The internal clock: Evidence for a temporal oscillator underlying time perception with some estimates of its characteristic frequency. Perception.

[CR87] van Rossum, G. (2020). *The Python library reference* (Release 3.8.2) [Computer software]. Python Software Foundation.

[CR88] van Rossum, G., & Drake Jr., F. L. (1995). *Python reference manual*. *Centrum Voor Wiskunde En Informatica Amsterdam.*

[CR89] von der Malsburg, T. (2015). Saccades: An R package for detecting fixations in raw eye tracking data (Version 0.1) [Computer software]. *Zenodo*. Advance online publication. 10.5281/zenodo.31799

[CR90] Walsh V (2003). A theory of magnitude: Common cortical metrics of time, space and quantity. Trends in Cognitive Sciences.

[CR91] Whitaker MM, Hansen RC, Creem-Regehr SH, Stefanucci JK (2022). The relationship between space and time perception: A registered replication of Casasanto and Boroditsky (2008). Attention, Perception, & Psychophysics.

[CR92] Wickham H (2007). Reshaping data with the reshape package. Journal of Statistical Software.

[CR93] Wickham, H. (2009). *Ggplot2: Elegant Graphics for Data Analysis* (2nd). Springer Publishing Company, Incorporated.

[CR94] Wickham, H., François, R., Henry, L., & Müller, K. (2018). dplyr: A grammar of data manipulation (R package Version 1.0.0) [Computer software]. https://CRAN.R-project.org/package=dplyr

[CR95] Winter B, Marghetis T, Matlock T (2015). Of magnitudes and metaphors: Explaining cognitive interactions between space, time, and number. Cortex.

