# Supplementary material for: Tau and kappa in interception – how perceptual spatiotemporal interrelations affect movements
Source: Atten Percept Psychophys. 2022 Jun 15;84(6):1925–43. doi: 10.3758/s13414-022-02516-0 (PMC9338162; doi:10.3758/s13414-022-02516-0)
Supplement: Supplementary file 1 — (DOCX 308 kb) [file 13414_2022_2516_MOESM1_ESM.docx]

Online Supplement

Article Title: Tau and kappa in interception – how perceptual spatiotemporal interrelations affect movements

Journal name: Attention, Perception & Psychophysics

Authors names:

Anna Schroeger^a^*

Markus Raab^b,c^

Rouwen Cañal-Bruland^a^*

^a^Department for the Psychology of Human Movement and Sport, Institute of Sport Science, Friedrich Schiller University Jena, Germany

^b^Department of Performance Psychology, Institute of Psychology, German Sport University Cologne, Germany

^c^School of Applied Sciences, London South Bank University, UK

*Correspondence concerning this article should be addressed to Anna Schroeger (annaschroeger@gmail.com).

**EXPERIMENT 1**

# Results

## Interception data

Outlier analyses led to 0.06-1.85% data exclusion (see Table S1).

*Table S1*. Excluded interception data due to outlier correction.

|  | Experiment 1 | | Experiment 2 | |
| --- | --- | --- | --- | --- |
| Outlier exclusion | visual | auditory | visual | auditory |
| spatial response | 0.41% | 0.06% | 0.44% | 0.02% |
| temporal response | 0.84% | 1.85% | 1.00% | 1.27% |

## Post-hoc analyses

To test whether task difficulty might explain the absent typical kappa effect in the visual domain, post-hoc the spatial and temporal error scores per task modality were plotted (see Figure S1). Indeed, the temporal responses were very similar for the auditory and the visual conditions, whilst the variation of the spatial responses was clearly higher in the auditory condition. If variability depicts uncertainty or noise, this may explain why in the auditory condition, there was an effect of the temporal context (low noise) on the spatial response (high noise), whereas in the visual condition the spatial context (low noise) did not affect the temporal response (low noise).


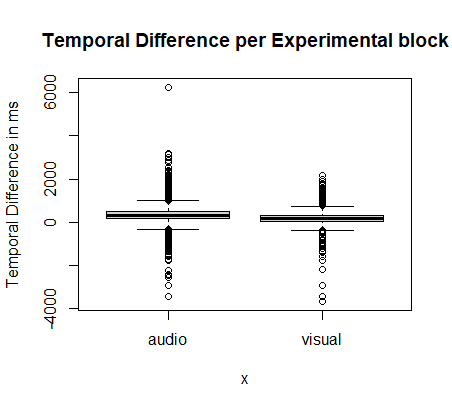

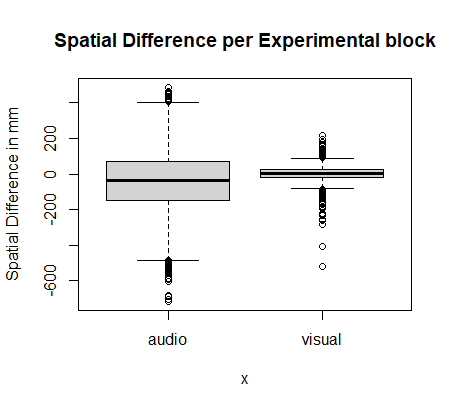


Figure S1. Descriptive data. Left: Temporal difference between the correct time and participants responses per condition. Right: Spatial difference between correct location and participants’ interception location.

Additionally, in an exploratory analysis we tested the representational noise hypothesis which states that more noise in the dependent variable will lead to larger biases of the manipulation. To this end, we inspected the individual effects per participant (kappa and tau) per modality (auditory vs. visual; see Figure S2). For all except the auditory tau effect, participant’s effect sizes were relatively similar (narrow distribution). Therefore, we further analyzed the auditory tau effect, by correlating the individual size of the tau/kappa effects with the visual-relative-to-auditory reliability (indicated as quotient of variances) in localization (see Figure S3). There was no significant relation, r = .211, p = .181.


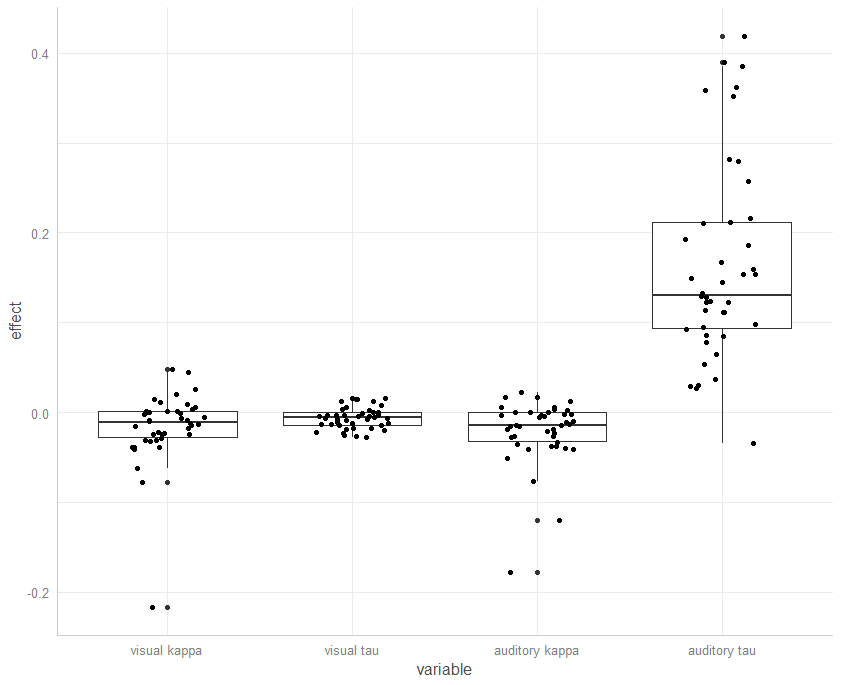


Figure S2. Individual effect sizes for kappa and tau effects per sensory modality.


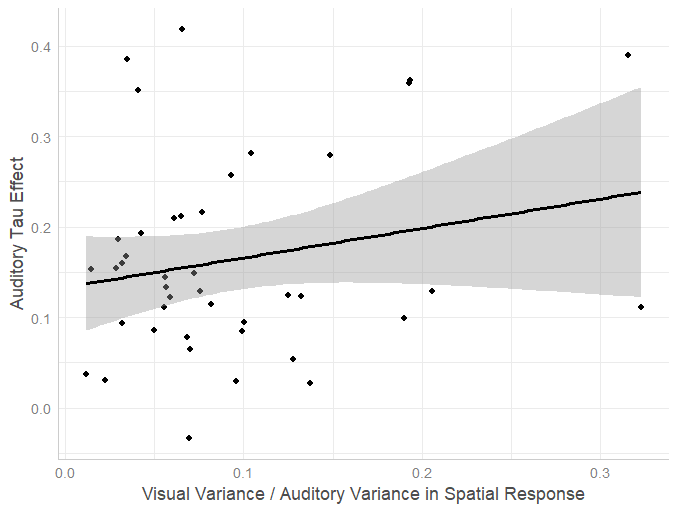


Figure S3. Relationship between the auditory tau effect and the reliability of visual compared to auditory input in localization (quotient of variances). Higher values represent higher visual compared to auditory noise.

For auditory stimuli, volume only descriptively impacted the spatial and temporal variability, indicating that task difficulty did not increase with decreasing volume (Figure S4). Figure S5 shows that indeed the spatial error was more variable in the blurred condition, indicating that spatial localization was more difficult for blurred stimuli, but also the temporal error was affected.


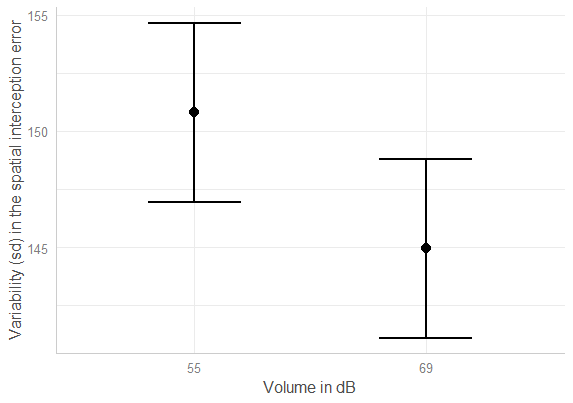

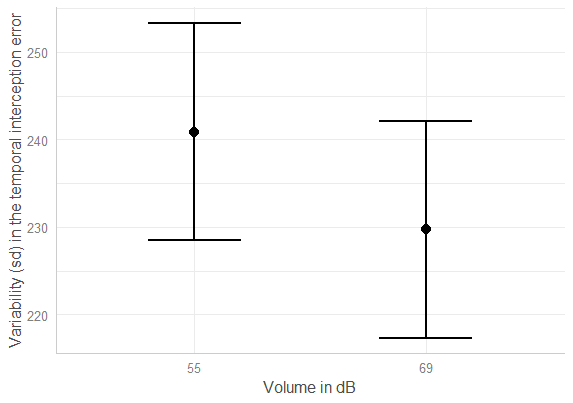


Figure S4. Variability of the spatial (left) and temporal (right) interception error per volume level. Depicted is the mean (dot) and the within-participant variability.


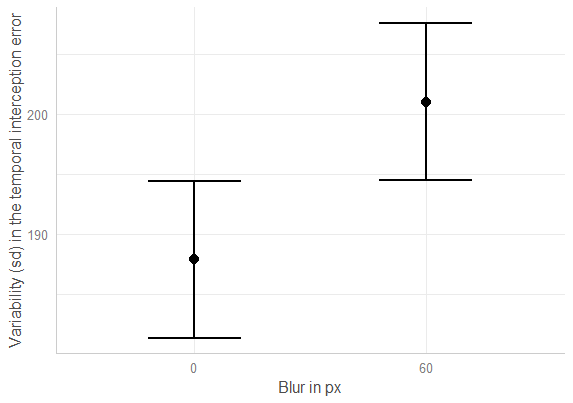

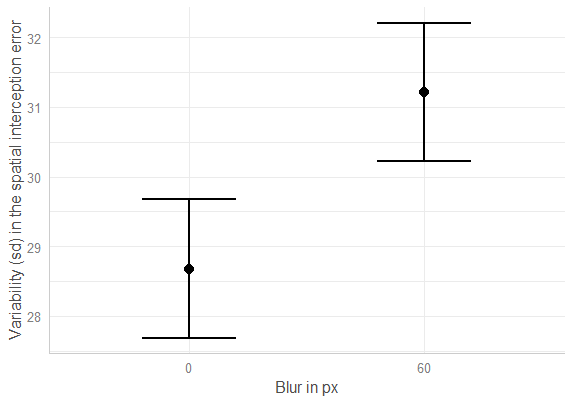


Figure S5. Variability of the spatial (left) and temporal (right) interception error per blur level. Depicted is the mean (dot) and the within-participant variability.

**EXPERIMENT 2**

# Results

## Interception data

Due to outlier exclusion, 0.02-1.27% of the data was rejected for analysis (see Table 1 in the Online Supplement).


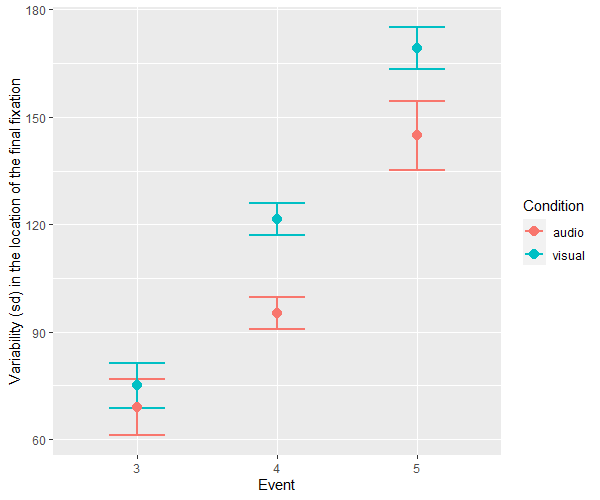


Figure S6. Variability of the gaze location per event. Depicted is the mean (dot) and the confidence intervals in the auditory and the visual condition.


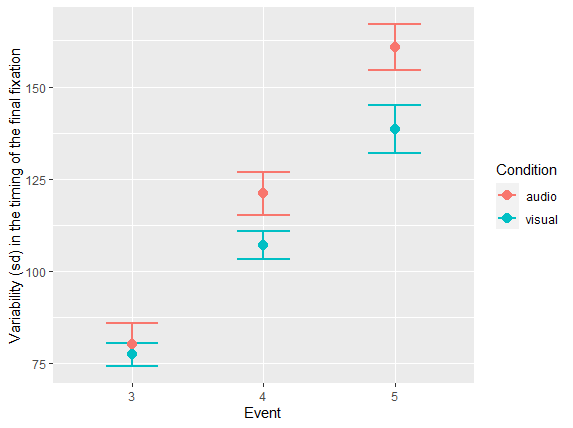


Figure S7. Variability of the initiation of the final fixation per event. Depicted is the mean (dot) and the confidence intervals in the auditory and the visual condition.

## Gaze data

Results of the fixation at the moment of interception revealed no visual tau effect (see Figure S8).


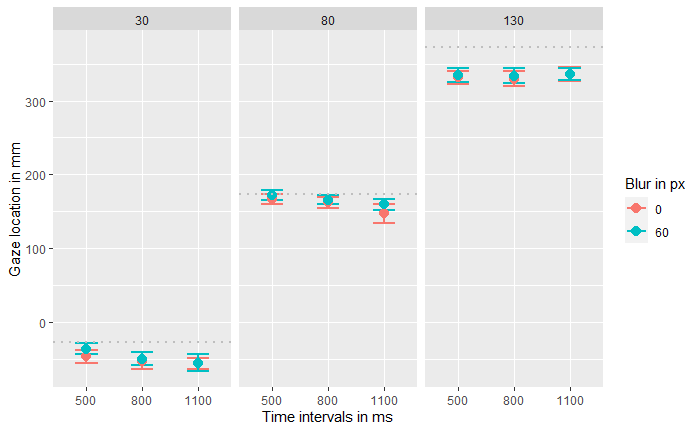


Figure S8. Visual tau effect (effect of temporal intervals on the gaze location at the moment of tapping on the screen). One plot for each spatial interval is displayed. Depicted is the mean (dot) and the within-participant variability in the auditory and the visual condition.

Aglioti, S., DeSouza, J. F., & Goodale, M. A. (1995). Size-contrast illusions deceive the eye but not the hand. *Current Biology*, *5*(6), 679–685. https://doi.org/10.1016/S0960-9822(95)00133-3

Cañal-Bruland, R., Voorwald, F., Wielaard, K., & van der Kamp, J. (2013). Dissociations between vision for perception and vision for action depend on the relative availability of egocentric and allocentric information. *Attention, Perception, & Psychophysics*, *75*(6), 1206–1214. https://doi.org/10.3758/s13414-013-0476-3

de la Malla, C., Brenner, E., Haan, E. H. F. de, & Smeets, J. B. J. (2019). A visual illusion that influences perception and action through the dorsal pathway. *Communications Biology*, *2*, 38. https://doi.org/10.1038/s42003-019-0293-x

de la Malla, C., Smeets, J. B. J., & Brenner, E. (2018). Errors in interception can be predicted from errors in perception. *Cortex*, *98*, 49–59. https://doi.org/10.1016/j.cortex.2017.03.006

Franz, V. H., Gegenfurtner, K. R., Bülthoff, H. H., & Fahle, M. (2000). Grasping Visual Illusions: No Evidence for a Dissociation Between Perception and Action. *Psychological Science*, *11*(1), 20–25. https://doi.org/10.1111/1467-9280.00209

Goodale, M. A., & Milner, A. D. (1992). Separate visual pathways for perception and action. *Trends in Neurosciences*, *15*(1), 20–25. https://doi.org/10.1016/0166-2236(92)90344-8

Goodale, M. A., Milner, A. D., Jakobson, L. S., & Carey, D. P. (1991). A neurological dissociation between perceiving objects and grasping them. *Nature*, *349*(6305), 154–156. https://doi.org/10.1038/349154a0

Haffenden & Goodale (1998). The effect of pictorial illusion on prehension and perception. *The Brain & Neural Networks*, *5*(2), 78_2-79. https://doi.org/10.3902/jnns.5.78_2

Medendorp, W. P., Brouwer, A. J. de, & Smeets, J. B. J. (2018). Dynamic representations of visual space for perception and action. *Cortex*, *98*, 194–202. https://doi.org/10.1016/j.cortex.2016.11.013
